# The birth of Perspectives on Medical Education

**DOI:** 10.1007/s40037-012-0001-8

**Published:** 2012-02-07

**Authors:** Jan C. C. Borleffs

**Affiliations:** University Medical Center Groningen and University of Groningen, Groningen, the Netherlands

Medical education has a long history in the Netherlands and Flanders. This is not only reflected by the leading role of Dutch and Flemish universities in both undergraduate and postgraduate training, but also by the high number of scientific articles in international journals. A few examples to support these introductory statements.

The national Blueprint with objectives of undergraduate medical education was first published in 1994 and contained one of the first descriptions of medical programmes in the world [1]. Soon after its publication, the Dutch Ministry of Health introduced the Blueprint as the nationwide ‘gold standard’ of medical education to be used for accreditation purposes. In 2009 the third edition of the Blueprint was published, referring to the CanMEDS competency framework as the basis for medical undergraduate training [2]. Furthermore, in the postgraduate medical specialist training, Dutch and Flemish universities appear to be enthusiastic followers of modernization task forces. The process of modernization according to the CanMEDS model is so energetic in the Netherlands and Flanders that its implementation is only a few steps behind that of our Canadian colleagues!

With respect to scientific research in the area of medical education, the Netherlands is among the most active countries. In a bibliometric survey of the number of publications in international peer-reviewed journals in 2010 the Netherlands ranked number 3 after Canada and the UK. Furthermore, the list of most productive authors is led by Dutch colleagues [3]. Given the size of the country and the relatively small number of medical schools (8 in the Netherlands and 5 in Flanders), these figures characterize the prominent role of Dutch research groups in the field of medical education.

Finally, there is the very active Netherlands Association of Medical Education (Nederlandse Vereniging voor Medisch Onderwijs, NVMO) that has become a platform for discussion and for sharing experiences. In 2012 the NVMO will celebrate its 40th anniversary. The association’s 1,200 members represent an important group of clinical and preclinical health care professionals, educationalists, policymakers, students, and other professionals with an interest in health professions education.

With this history in mind, it is obvious that we had a journal for the Dutch-speaking countries, i.e., Tijdschrift voor Medisch Onderwijs (TMO, Netherlands Journal of Medical Education). TMO was first published in 1982 and developed into a peer-reviewed journal with six issues published annually. Because the journal was published in Dutch, however, opportunities to reach an internationally oriented audience were inevitably limited. This clearly constituted a barrier to broadening the scope of the journal and diminished its appeal to the increasingly internationally oriented health professions education community. Consequently, after 30 years of having published in Dutch, we have now reached the next step in the development of the journal, i.e., a transformation into an international English-language journal. Moreover, by transforming TMO into *Perspectives on Medical Education* we will be able to offer international authors an additional option for publication. Although we are aware that the number of medical education journals is quite substantial, we believe that this new international journal will have added value, providing Dutch medical education a place in the international arena, and thereby widening the views of both our Dutch and our international readers.

PME aims to provide an international platform for innovation and research in health professions education. Submissions that address a wide range of topics—innovation and research in medical education, case studies, practical guidelines, opinion papers—are welcome.

Not only the language and the title have changed. Starting in January 2012 the administrative and editorial process of the journal has been taken over by our publisher Bohn Stafleu van Loghum, which is part of Springer Media. Consequently, articles will be published electronically soon after acceptance for publication (free access) and can be found through the PubMed search system. It also means that we have to say goodbye to Geertje Karsten–van der Giessen, who supported the Editorial Board as a secretary of the journal for many years, and Mereke Gorsira, who helped us with the English summaries of the articles of TMO. Both have made an important contribution to the journal, which we highly appreciate.

For this first issue of PME the Editorial Board invited international authors who have a link with the Netherlands or Flanders. They either worked or spent a sabbatical in the Netherlands or they have a history of close collaboration with colleagues in the Netherlands or Flanders. All articles of this first issue deal with a specific topic in medical education, some of which by reviewing a general theme, others by referring to a theme of which the development has been promoted by researchers in the Netherlands and Flanders.

We are convinced that this first issue of *Perspectives on Medical Education* will offer you a handful of interesting topics. Furthermore, we hope that this issue will inspire you to submit manuscripts to PME.
